# A New Azaphilone, Kasanosin C, from an Endophytic *Talaromyces* sp. T1BF

**DOI:** 10.3390/molecules15063993

**Published:** 2010-06-01

**Authors:** Liang-Qun Li, Yan-Guang Yang, Ying Zeng, Cheng Zou, Pei-Ji Zhao

**Affiliations:** 1 The State Key Laboratory of Phytochemistry and Plant Resources in West China, Kunming Institute of Botany, Chinese Academy of Sciences, Kunming 650204, China; 2 Kunming Medical University, Kunming 650031, China; 3 Guiyang Medical University, Guiyang 550004, China

**Keywords:** endophytic, *Talaromyces*, *Taxus yunnanensis*, azaphilone

## Abstract

The strain T1BF was isolated from the old bast tissue of *Taxus yunnanensis* and determined to be a member of *Talaromyces*. The extracts from the solid fermentation of *Talaromyces* sp. T1BF were purified and obtained three azaphilones, including a new one. They were identified on the basis of spectral data as 6α-hydroxy-7β-methyl-8-oxo-3-((*E*)- prop-1-en-1-yl)-5,6,7,8-tetrahydro-1*H*-isochromen-7-yl-4'-hydroxy-2'-methoxy-6'-methyl- benzoate, named as kasanosin C (**1**), entonaemin A (**2**) and (+)-mitorubrin (**3**).

## 1. Introduction

**Figure 1:**
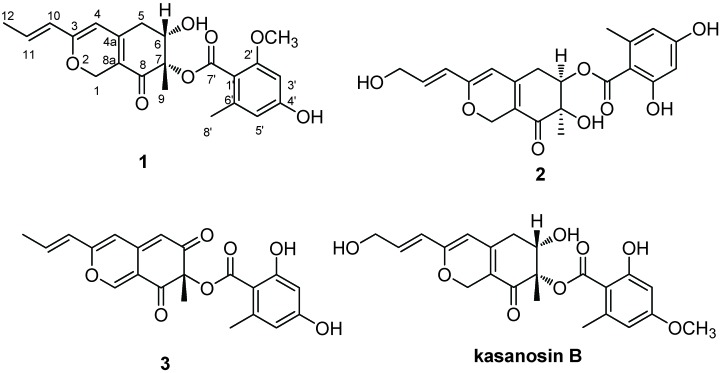
Structures of compounds **1**-**3** and kasanosin B.

Endophytes commonly present in almost all plants are well-known as sources of bioactive secondary metabolites [1,2], e.g. a novel taxol-producing endophytic fungus was discovered in *Taxus brevifolia* [1]. *Taxus yunnanensis* Loes. (Taxaceae), which mostly distributed in the southwest of China, is well recognized for producing anticancer taxoid compound [3]. In our experiments searching for new compounds from endophytic microorganisms, a series of novel compounds were obtained [4,5,6]. During an ongoing search for new bioactive metabolites from plant endophytic microorganisms, an isolate of *Talaromyces* sp. T1BF, obtained from the old bast tissue of *Taxus yunnanensis* was investigated. Herein, we describe the isolation and structural elucidation of three compounds, including a new azaphilone, from T1BF (Figure 1).

## 2. Results and Discussion

Compound **1** was obtained as a yellow amorphous solid. The HR-ESI-MS data indicated a molecular formula of C_22_H_24_O_7_ based on the [*M* + Na]^+^ ion signal at *m/z* 423.1424 (calc. 423.1419). The NMR data (Table 1) revealed ten quaternary carbons including two carbonyl groups (δ 193.0 and 169.5), and six methines, two methylenes and four methyl groups including one methyl connected to oxygen, which suggested compound **1** was an azaphilone [7]. Compound **1** exhibited the ^1^H and ^13^C data similar to those of kasanosin B [7], except that the C-12 which was a methyl group in compound **1**, which was oxygen-connected methylene in kasanosin B, and the methoxyl substituent at the benzoate unit was located at a different position (Figure 1). The structure of **1** was confirmed by detailed HMQC and HMBC experiments (Table 1). H-6 in compound **1** appeared as a doublet of doublets with *J* values of 8.4 and 4.8 Hz, while that in compound **2** was observed as a triplet with a small *J* value of 3.2 Hz. These data indicated that H-6 in compounds **1** and **2** was located at different orientation. Moreover, H-6 in **1** might be placed in an axial position, since H-6 only has a correlation with H-5β, in its ROESY spectrum. And a NOESY experiment showed a NOE interaction between H-6 and H-9 supporting the proposed relative configuration. Based on all the above data, compound **1** was identified as 6α-hydroxy-7β-methyl-8-oxo-3-((*E*)-prop-1-en-1-yl)-5,6,7,8-tetrahydro-1H-isochromen- 7-yl-4'-hydroxy- 2'-methoxy-6'-methylbenzoate, named as kasanosin C (Figure 1). Compounds **2**-**3** were determined as entonaemin A (**2**) [8] and (+)-mitorubrin (**3**) [9,10] by comparison with the data given in the corresponding references.

**Table 1:** NMR data of compounds **1~3**.

Position	1^a^			2^a^		3^b^	
	^1^H	^13^C	HMBC	^1^H	^13^C	^1^H	^13^C
1	4.88 (d, 14.0)	64.7	3, 4a, 8	5.01 (d, 10.9)	64.7	8.11 (s)	155.0
	4.80 (d, 12.8)		3, 4a, 8	4.80 (d, 12.8)			
3	-	161.1	-	^-^	161.5	-	156.4
4	5.43(s)	104.4	3, 5, 8a, 10	5.51, s	105.7	5.59 (s)	101.6
4a	-	149.7	-	-	149.2	-	144.0
5	2.83 (dd, 6.6, 18.0)	34.6	4, 4a, 6, 7, 8a	3.09 (brd, 19.2)	32.9	6.27 (s)	109.3
	2.61 (dd, 4.8, 17.6)		4, 4a, 6, 7, 8a	2.80 (brd, 19.2)			
6	4.00(dd, 4.8, 8.4)	73.9	4a, 5, 7, 8, 9	5.59 ( t, 3.2)	78.3	-	193.1
7	-	86.0	-	-	75.5	-	86.6
8	-	193.0	-	-	197.0	-	192.0
8a	-	116.3	-	-	115.5	-	115.7
9	1.67(s)	16.7	6, 7, 8	1.42, s	23.6	1.64 (s)	23.8
10	6.02 (dd, 13.2, 1.2)	126.0	3, 12	6.22 (dt, 15.6, 1.6)	123.2	6.27 (dt, 15.6, 1.6)	123.5
11	6.46 (dq, 14.0, 6.8)	134.8	3, 12	6.55 (dt, 15.5, 4.6)	138.4	6.63 (dq, 15.5, 3.6)	135.8
12	1.85 (dd, 1.2, 6.8)	18.4	10, 11	4.21 (brd, 3.2)	62.6	1.93 (d, 1.4)	18.5
1'	-	116.3	-	^-^	105.7	^-^	107.9
2'	-	159.7	-	-	166.4		166.0
3'	6.24 (d, 1.6)	97.5	1', 4', 5', 7' (w)	6.12(s)	101.7	6.21 (s)	101.6
4'	-	160.9	-	^-^	164.0	^-^	163.9
5'	6.17 (d, 2.0)	109.8	1', 3' , 4', 8'	6.12(s)	112.6	6.12 (s)	112.5
6'	-	139.0	-	^-^	144.9	^-^	144.7
7'	-	169.5	-	^-^	172.1	^-^	170.5
8'	2.15 (s)	19.4	1', 5', 6', 7' (w)	2.21, s	24.5	2.44 (3H, s)	22.7
2'-OCH_3_	3.68 (3H, s)	56.1	2'				

^a^in CD_3_OD; ^b^in CD_3_OCD_3_

## 3. Experimental

### 3.1. General

UV spectra were measured on a Shimadzu UV-2401PC spectrophotometer, *λ*_max_ (log *ε*) in nm. NMR experiments were carried out on Bruker AM-400 and Bruker DRX-500 NMR spectrometers with TMS as internal standard. ESI-MS and HR-ESI-MS were recorded on a Finnigan LCQ-Advantage mass spectrometer and a VG Auto-Spec-3000 mass spectrometer. Optical rotations were measured on a Jasco DIP-370 digital polarimeter. Column chromatography was carried out on silica gel (G, 200-300 mesh and H, Qingdao Marine Chemical Factory, Qingdao, PR China), Sephadex LH-20 (Pharmacia), reverse-phase C_18_ (RP-18) silica gel (Merck) and MCI gel (Mitsubishi Chemical Corporation, Tokyo, Japan). Thin-layer chromatography (TLC) was performed on silica gel (Si gel G; Qingdao Marine Chemical Factory, Qingdao, China). Solvents were of the industrial purity and distilled prior to use.

### 3.2. Microbial material

The old bast of *Taxus yunnanensis* was collected at Kunming Botanic Garden, Kunming Institute of Botany, Chinese Academy of Sciences, Yunnan, P. R. China, in August 2008. The plant materials were washed under running tap water and were sterilized successively with 75% ethanol for 1 min and 0.1% mercury perchloride for 5 min, then rinsed five times in sterile water and cut into small pieces which were incubated at 25 ^o^C on YMG media (yeast extract 4.0 g, malt extract 10.0 g, glucose 4.0 g, agar 15.0 g, distilled water 1,000 mL) and cultured until colony or mycelium appeared surrounding the segments. After culturing about one mouth, a fungal strain named T1BF appeared, which was isolated from the sterilized bast, and identified as *Talaromyces* by Prof Liu Yun-Long (Yunnan Agricultural University). A sample is deposited at the Kunming Institute of Botany.

### 3.3. Extraction and isolation

The strain was cultured in 10 L of PDA medium [consisting of potato (200 g/L), dextrose (20 g/L), and agar (15 g/L)]. After cultivation for two weeks at 28 ^o^C, the cultures were exhaustively extracted five times with AcOEt/MeOH/AcOH (80:15:5) to obtained extract (39 g). The extract was chromatographed on silica gel (silica gel G, 150 g) and eluted with petroleum ether/acetone (10:1 to 7:3) to afford two fractions (PE-1 to PE-2), and eluted with CH_3_Cl/MeOH (20:1 to 8:2) afford five fractions (CH-1 to CH-5). Fraction PE-1 (215 mg) was applied on Sephadex LH-20 and eluted with methanol to provide fractions PE-1-1 and PE-1-2. Fraction PE-1-1 (190 mg) was purified on silica gel G (7 g) eluting with CH_3_Cl/acetone (10:1 to 8:2) to obtain compound **3** (2.2 mg). PE-2 (296 mg) was subjected to MCI-gel column chromatography (50 g) eluting with acetone/water (1:1 to 9:1) to afford two fractions (PE-2-1 and PE-2-2). PE-2-1 (100 mg) was subjected to Sephadex LH-20 chromatography eluting with CH_3_Cl/MeOH (1:1) and then was chromatographed on silica gel H (5 g) eluting with petroleum ether/acetone (6:1 to 2:1) to obtain compound **2** (10 mg). PE-2-2 (60 mg) was subjected to Sephadex LH-20 chromatography eluting with MeOH and then chromatographed on silica gel H (10 g) eluting with CH_3_Cl/acetone (100:1) to obtain compound **1** (3 mg).

*Kasanosin C* (**1**): Yellow amorphous solid. 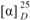 : = + 151 (*c* = 0.13, MeOH). UV (MeOH) λ_max _(log ε): 205 (5.30), 284 (2.39), 374 (3.66). ^1^H and ^13^C-NMR data (500 and 125 MHz, resp.) see Table 1. ESI-MS: 423 [*M* + Na]^+^; HR-ESI-MS: 423.1424 [*M* + Na]^+ ^(calc. 423.1419).

*Entonaemin A* (**2**): Yellow powder. 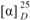: = + 132 (*c* = 0.32, MeOH). ^1^H and ^13^C-NMR data (400 and 100 MHz, resp.) see Table 1. ESI-MS: 425 [*M* + Na]^+^. 

*(+)-Mitorubrin* (**3**): Yellow powder. 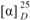: = 144 (*c* = 0.13, acetone). ^1^H and ^13^C-NMR data (500 and 125 MHz, resp.) see Table 1. ESI-MS: 405 [*M* + Na]^+^.

## 4. Conclusions

A new azaphilone as well as two known azaphilones were isolated from an endophytic *Talaromyces* fungus sp. The new compound was determined to be kasanosin C by spectroscopic methods, including HR-ESI-MS and 2D-NMR experiments, in combination to the comparison with known compounds. 
